# Complete Genome Sequence of the Lysogenic Pseudomonas Bacteriophage Fyn8

**DOI:** 10.1128/mra.00004-23

**Published:** 2023-02-13

**Authors:** Magnus Z. Østergaard, Andreas S. Damgaard, Elisabeth Rosenbæk, Thøger J. Krogh, Clare L. Kirkpatrick

**Affiliations:** a Department of Biochemistry and Molecular Biology, University of Southern Denmark, Odense, Denmark; b Amplexa Genetics A/S, Odense, Denmark; Queens College Department of Biology

## Abstract

A temperate bacteriophage infecting Pseudomonas aeruginosa PAO1 was isolated from river water. Nanopore sequencing revealed that it has a circular double-stranded DNA genome of 45,617 bp, containing typical phage structural proteins and lambda-like lysogeny regulators. Putative O-antigen serotype conversion and anti-cyclic oligonucleotide-based antiphage signaling system (CBASS) defense system proteins were also identified.

## ANNOUNCEMENT

Pseudomonas aeruginosa is an opportunistic pathogen that has been the subject of various phage therapy trials but with mixed success (1–3). Environmental samples are a potential source of novel phages that could contribute to improvements in this field. Here, we describe the genome sequence of a bacteriophage that was isolated from river water in Odense, Denmark, and enriched on Pseudomonas aeruginosa PAO1 according to standard protocols (4). River water was centrifuged, filtered, mixed 1:1 with 2× LB medium, and inoculated with a 1/4,000 volume of an overnight culture of PAO1. After 12 h of incubation at 37°C, the culture was centrifuged, and the supernatant was filtered. Diluted supernatant was plated on PAO1, and Fyn8 was purified from a single plaque. Lysates were prepared on PAO1 lawns, and bacteriophage DNA was extracted with the Qiagen DNeasy blood and tissue kit. DNA was neither sheared nor size selected before library preparation.

DNA was prepared for sequencing with the Nanopore rapid barcoding kit (SQK-RBK004). Sequencing was performed with a Nanopore FLO-MIN106 flow cell (flow cell serial number, FAU83294; device serial number, MN36371). Base calling was performed using Guppy base-calling software v6.1 (Oxford Nanopore Technologies [ONT]) with minimap2 v2.22-r1101. The high-accuracy base-calling model (dna_r9.4.1_450bps_hac.cfg) with a default minimum Q score of 8 was used. We obtained 21,268 reads, with a read *N*_50_ value of 6,455 bp and a median read quality score of 11.8. These reads were filtered for quality using Filtlong v0.2.1 (https://github.com/rrwick/Filtlong), set at 95% of 1-kbp reads retained. The remaining 5,178 reads were assembled into a single circular contig with 589-fold mean coverage with Flye v2.9.1-b1780 (5), with default settings, a genome size of 0.03 Mbp, and minimum coverage of 50×, and further polished with Medaka v1.0.3 (https://github.com/nanoporetech/medaka), with default settings and the r941_min_high_g360 model, to construct the final genome. Automatic annotations were performed using the Bacterial and Viral Bioinformatics Resource Center (BV-BRC) v3.27.0 annotation service bacteriophage pipeline (5), which is based on PHANOTATE v1.5.0 (6). The results were refined with additional functional predictions using InterProScan v5.91-91 (7) and BLASTx (8). We could not assign terminus positions with PhageTerm because the library preparation method and the long but few reads of our ONT sequencing output are incompatible with this software (9). However, read mapping revealed a uniform distribution across the assembly, consistent with lack of defined termini. We also obtained three single reads with greater-than-genome length, the ends of which are repeats mapping to different regions for each read, suggesting that the genome of Fyn8 is most likely circularly permuted. We acknowledge that genomes assembled solely from ONT data may have limitations and that hybrid Illumina-ONT sequencing would provide highest-quality data.

The genome of Fyn8 is 45,617 bp, with a GC content of 59.9%. A BLASTn search with the whole genome showed that the first ~20,000 bp, starting from the terminase and containing primarily bacteriophage structural genes, was 94% identical to bacteriophages phi297 (10) and ctwxn6 (11), while the remainder of the genome did not continuously align with any other sequence. The genome contains 66 protein-coding genes, of which 22 could be assigned a predicted function, and 2 tRNAs.

### Data availability.

The genome sequence of Fyn8 was deposited in GenBank under accession number OP966821. The raw sequence reads were deposited in SRA under accession number PRJNA914104.
